# Assessment of Metabolic and Nutritional Imbalance in Mechanically Ventilated Multiple Trauma Patients: From Molecular to Clinical Outcomes

**DOI:** 10.3390/diagnostics9040171

**Published:** 2019-11-01

**Authors:** Alexandru Florin Rogobete, Ioana Marina Grintescu, Tiberiu Bratu, Ovidiu Horea Bedreag, Marius Papurica, Zorin Petrisor Crainiceanu, Sonia Elena Popovici, Dorel Sandesc

**Affiliations:** 1 Faculty of Medicine, “Victor Babes” University of Medicine and Pharmacy, Timisoara 300041, Romania; alexandru.rogobete@umft.ro (A.F.R.); bedreag.ovidiu@umft.ro (O.H.B.); marius.papurica@gmail.com (M.P.); zcrainiceanu@gmail.com (Z.P.C.); dsandesc@yahoo.com (D.S.); 2 Clinic of Anaesthesia and Intensive Care, Emergency County Hospital “Pius Brinzeu”, Timisoara 300723, Romania; popovici.sonia@yahoo.com; 3 Faculty of Medicine, “Carol Davila” University of Medicine and Pharmacy, Bucharest 020021, Romania; ioana.grintescu@rospen.ro

**Keywords:** indirect calorimetry, nutrition, metabolism, trauma, intensive care unit

## Abstract

The critically ill polytrauma patient is characterized by a series of metabolic changes induced by inflammation, oxidative stress, sepsis, and primary trauma, as well as associated secondary injuries associated. Metabolic and nutritional dysfunction in the critically ill patient is a complex series of imbalances of biochemical and genetic pathways, as well as the interconnection between them. Therefore, the equation changes in comparison to other critical patients or to healthy individuals, in which cases, mathematical equations can be successfully used to predict the energy requirements. Recent studies have shown that indirect calorimetry is one of the most accurate methods for determining the energy requirements in intubated and mechanically ventilated patients. Current research is oriented towards an individualized therapy depending on the energy consumption (kcal/day) of each patient that also takes into account the clinical dynamics. By using indirect calorimetry, one can measure, in real time, both oxygen consumption and carbon dioxide production. Energy requirements (kcal/day) and the respiratory quotient (RQ) can be determined in real time by integrating these dynamic parameters into electronic algorithms. In this manner, nutritional therapy becomes personalized and caters to the patients’ individual needs, helping patients receive the energy substrates they need at each clinically specific time of treatment.

## 1. Introduction

The critically ill polytrauma patient represents a very complex, multifactorial case of associated pathologies that significantly increase mortality rates [1,2,3]. Both primary trauma and secondary, post-traumatic injuries lead to a worsening of the clinical and biological status of these patients, presenting a real challenge for intensive care units (ICUs) [4,5]. Among these complications, the most important are systemic inflammatory response syndrome (SIRS) [6,7], cardiogenic shock, sepsis [8], acute respiratory distress syndrome (ARDS) [9,10,11], ventilator-associated complications, oxidative stress (OS) [12,13,14], and malnutrition [15]. When looking at these issues objectively and from a molecular point of view, the critically ill patient’s nutrition status is closely related to all the above-mentioned complications. A high degree of malnutrition or an inadequate nutrition strategy in the case of critically ill polytrauma patients can significantly increase the rate of secondary, post-traumatic complications, leading to a vicious cycle in which inflammation, the immune system, infections, and increased oxygen consumption are strongly interconnected [16].

In an international multicenter study, Heyland et al. reported that over 70% of the patients included in the study did not receive the minimum 80% of their energy requirements [17]. They showed, at a global level, that a high percentage of critical patients admitted to the ICU suffer from underfeeding, and, consequently, are malnourished [17]. The mechanisms that initiate malnutrition in critically ill polytrauma patients are multifactorial, depending on a series of molecular, genetic, cellular, and clinical factors [18,19,20]. The most common are represented by an increase in oxygen requirements, aberrant increase of metabolism, coagulation and immune system imbalances, infections, and inadequate administration of nutritional therapy [7,21,22].

For decades, clinicians have been using mathematical equations to predict energy requirements. However, numerous studies have reported a disparity between the clinical status of the patient and the values rendered by these calculations, especially in the case of critically ill patients. A recent study published by Zusman et al. concluded that mathematical equations cannot replace the indirect calorimetry monitoring of energy requirements for critical patients. This study included a high number of patients, with over 3500 quantifications [23].

Regarding the metabolic disorders of critically ill polytrauma patients, some of the most important clinical symptoms are hyperglycemia and increased insulin resistance, rapid loss of muscular mass, and disturbance of the nitrogen balance [24,25]. The imbalances produced in the lipid profile by the augmentation of pro-oxidative stress are important characteristics that strongly influence the metabolic status of these patients. When looking at the symptoms as a whole, nutrition should be considered to be one of the most important therapeutic actions in the case of critically ill polytrauma patients. Recent studies have shown important implications of nutrition both in the modulation of the patients’ responses to metabolic stress and in their clinical outcome [26,27].

In the literature, there are a series of controversies regarding the adjustment of nutrition in the case of critically ill polytrauma patients, as several studies present advantages and disadvantages for both enteral and parenteral nutrition [28,29,30]. On the one hand, it has been reported that by using enteral nutrition there is a higher rate of infections and longer stays in intensive care for these patients. On the other hand, by administering parenteral nutrition, a series of adverse effects have been reported, such as hyperglycemia, augmentation of metabolic stress, and an increase in the incidence of sepsis.

The aim of this paper is to connect and present in a clinical setting the strong relationship between inadequate nutrition and a series of secondary complications, specific to the critically ill polytrauma patient. Furthermore, we wish to detail the modern methods used to determine energy expenditure, as well as new guidelines for adapting nutritional therapy in a specific manner to these patients.

## 2. Molecular and Pathophysiological Aspects of Metabolism

From a clinical point of view, the critically ill polytrauma patient is characterized by a series of primary traumatic injuries, as well as by a multitude of trauma-associated secondary injuries such as hemorrhagic shock, tissue hypoxia, generalized inflammation, oxidative stress, and infections. All of these subsequently lead to a multiple organ dysfunction syndrome (MODS) and to a significant increase in mortality rates [7,31,32,33,34].

**Figure 1:**
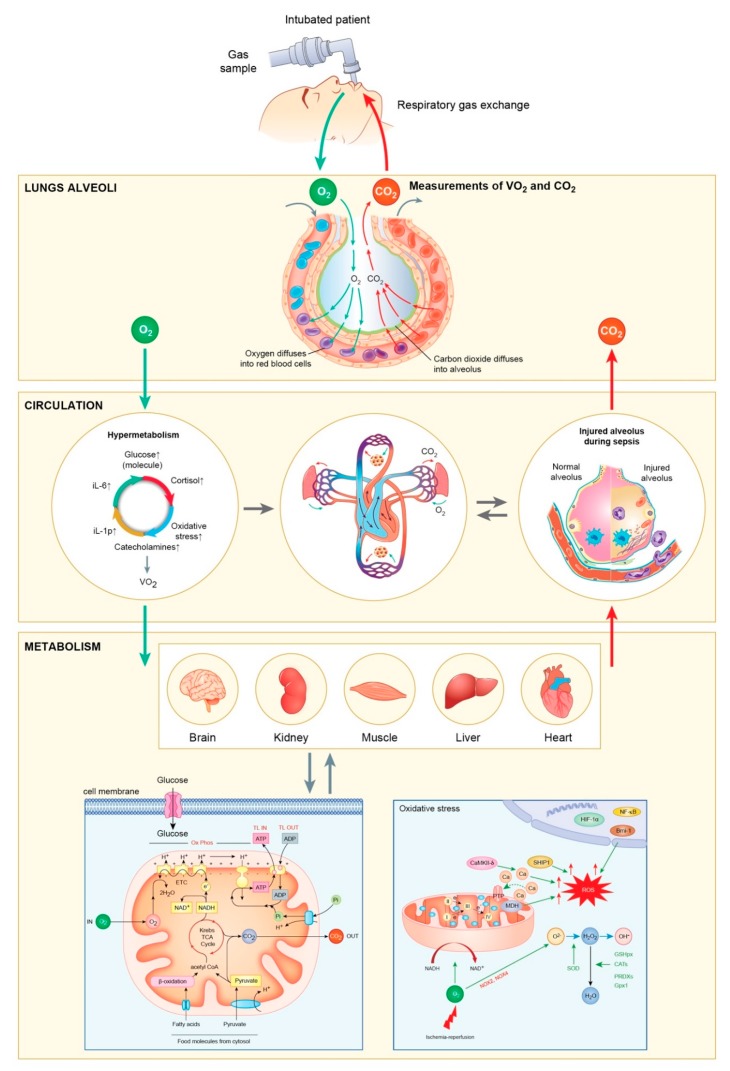
The entire metabolic process in the critical patient and the correlation with continuous gas exchange monitoring (VO_2_ and VCO_2_).

From a molecular point of view, the above-mentioned events lead to the activation of a series of molecular systems and mechanisms, such as coagulation [35,36,37,38], complement [39], fibrinolysis, and an immense quantity of pro- and anti-inflammatory mediators released from macrophages, granulocytes, and lymphocytes [5,9,40,41,42,43]. Among these, the most researched are interleukin 6 (IL-6), interleukin 8 (IL-8), interleukin 10 (IL-10), interleukin 17 (IL-17), and tumor necrosis factor alpha (TNF-α) (Figure 1) [40,41,42,43].

Redox balance is significantly affected, with significant quantities of free radicals being released. In this case, the metabolic imbalance is strongly affected at a molecular level, with the free radicals being involved in a series of protein and lipid denaturation reactions [12,44,45,46]. Moreover, the redox protein and lipid denaturation reactions lead to the release of other reactive species, leading to augmentation of the pro-oxidative cascade [16,47].

An important aspect in the pathophysiology of critically ill polytrauma patients is represented by the negative nitrogen balance [48,49,50]. One of the main responses to severe injury is represented by the accentuation of protein catabolism and by the loss of urinary nitrogen and phosphorus. The process of nitrogen loss is very complex, and recent studies have shown that it correlates significantly with a change in metabolic rate, with a maximum peak a few days after the injury, and a gradual return to baseline after a few weeks. Hence, it has been concluded that if the mobilization of amino acids from metabolized proteins is not rapidly corrected through adequate and personalized nutrition, the consequences are dramatic, manifested through a rapid loss of muscular mass, and a very long and difficult recovery [51,52,53].

Regarding the metabolic answer to trauma, a series of articles described the following three phases of the event: the ebb phase, the catabolic phase, and the anabolic phase [54,55,56]. One very important aspect is the different biological and biochemical characteristics of each phase. From a clinical point of view, each of these phases needs a different therapeutic intervention from a nutritional viewpoint. From a clinical point of view, the ebb phase lasts for 12 h to 36 h, depending on the severity of the injuries, whereas the flow phase lasts between seven days and three weeks [25,57,58,59]. A therapeutic decision with high accuracy cannot be made without adequate monitoring of the metabolic changes and associated energy requirements.

From a biochemical, molecular, and cellular point of view in trauma, we distinguish the ebb phase during the first 8 h to 24 h post-trauma, a phase characterized by important hemodynamic changes. From a clinical point of view, during this phase, volemic resuscitation through fluid and blood products is the basis of the therapy. Afterwards, during the next three days, patients are characterized by an aggressive production of cytokines and inflammatory molecules. During this phase, the metabolic disaster continues at a cellular level, with the redox imbalance being augmented and extensive. The last phase, described as an anabolic phase, is considered to be the one in which molecular and metabolic mechanisms are oriented towards recovery [25,60,61,62].

The existence of an inflammatory response without the clear presence of bacterial sources leads to an alarming activation of the immune system a short time after the traumatic event. These underlying signaling events are also called alarmines and influence the metabolic status of these patients considerably. The most researched endogenous signaling pathways responsible for the excessive augmentation of the immune response are represented by defensins, heat shock proteins (HSPs), cathelicidin, high-mobility group box 1 (HMGB1), and eosinophil-derived neurotoxin (EDN). Moreover, post-traumatic coagulopathy is responsible for a series of other side effects that lead to a metabolic imbalance [42,63,64,65,66].

In this manner, we can frame these molecular mechanisms in the so-called acute phase response, through which the liver’s protein synthesis is redistributed depending on the severity of trauma. In these situations, adapting the nutrition becomes an impossible task without adequate and correct monitoring of each individual patient.

Although enteral nutrition is recommended by the majority of clinical studies, it was concluded that in certain patients, the desired enteral nutrition cannot be ensured because of digestive intolerance. In the case of critically ill polytrauma patients, this aspect is present in the majority of the situations [26,67,68]. Fully enteral nutrition is impossible to achieve because of the intolerance manifested in the first three to five days post trauma. In this situation, parenteral nutrition should be applied. There are no clear guidelines regarding the exact time when the nutrition should be initiated and there have been important debates on the subject in the literature, but there are important differences between different guidelines on the topic in certain situations.

The European Society of Parenteral and Enteral Nutrition (ESPEN, www.espen.org) recommends early initiation of enteral nutrition, in the first 24 h after admission to the ICU. On the other hand, The Canadian Society for Nutritional Science (CSCN, www.nutritionalsciences.ca) recommends initiation of enteral nutrition in the 24–48 h interval after admission to the ICU.

A critically ill polytrauma patient with sepsis also presents with changes, as well as specific interactions caused by plasmatic cholesterol and proteins. Recent studies have shown that hypercholesterolemia can be an important index for negative prognosis in these patients, especially because of the complex interactions that cholesterol has with protein fragments in plasma. Moreover, it has been noticed that hypercholesterolemia interfaces with the augmentation of the systemic inflammatory response [69]. Chiarla et al. conducted a study in order to identify correlations between plasmatic cholesterol levels and a series of metabolic changes in septic patients. Following this study, they reported that there are strong correlations between hypercholesterolemia and changes in the expression of amino acids in plasma [70].

## 3. Biochemical and Pathophysiological Aspects of the Hypermetabolic Status

The critically ill polytrauma patient is characterized by a hypermetabolic status that is divided into different clinical time periods. From a pathophysiological and molecular point of view, hypermetabolism is represented by a complex line of interconnected biochemical reactions that have disastrous effects on the clinical outcome. This is a very serious situation not only because of the hypermetabolism characterizing the critical period of these patients but also by the fact that this hypermetabolic status can continue years after the trauma, leading to a delayed recovery for these patients [58,71,72,73]. The hypermetabolic status is characterized, in particular, by an alarming increase in oxygen consumption throughout the body. The hypermetabolism that characterizes the polytrauma patient is extremely complex and has not been described in detail until now. This is mainly because there is a high interdependence between a series of reactions specific to inflammation, infections, hormonal reactions, as well as pharmacological and pharmacodynamic interactions. At a cellular level, there is a similar trend [74,75,76].

## 4. Genetic and Epigenetic Expressions Associated with Hypermetabolism

Another system involved in the modulation of both the hypermetabolic response and the inflammatory response specific for critically ill patients is represented by the genetic and epigenetic expressions [77,78,79,80,81,82,83,84,85,86,87,88,89,90,91,92,93,94,95,96,97,98,99,100,101,102,103,104,105,106,107,108,109,110,111,112]. The most sensitive systems capable of a fast reaction to hypermetabolic changes are the microRNAs epigenetic species. From a structural point of view, the microRNAs are synthesized inside the cell. Their biosynthesis takes place in the cell nucleus where the RNA polymerase II attacks and codes for specific genes. These coding reactions lead to the formation of pri-microRNAs and the biochemical reactions that follow are represented by the attack of the RNAse III endonuclease (Drosha) on the pri-microRNA, leading to the formation of pre-microRNAs. This reaction is catalyzed by the DiGeorge syndrome critical region 8 (DGCR8) complex. After their formation, the pre-microRNAs species are transported through the exportin-5 transporting protein from the cell nucleus into the cytoplasm. Here, the RNAse III endonuclease (Dicer) and the RNA binding protein (TRBP) initiate new biochemical reactions leading to the formation of mature microRNAs that will be further exported under various forms, such as microvesicles, exosomes, and apoptotic bodies. One of the most useful and interesting facts is that microRNAs that are transported outside the cell can be used as specific biomarkers for certain cellular pathologies, as well as for molecular damage reactions specific to a certain disease [109].

A series of correlations between the clinical status and microRNAs expressions can be made regarding the hypermetabolism, inflammation, as well as the malnutrition, of the critically ill polytrauma patient, and therefore one can state that the nutritional status and metabolic imbalances found in these patients can be evaluated by using this method. The critically ill polytrauma patient is especially characterized by an accentuated catabolic stress. Under these circumstances, skeletal muscle is one of the major sites for the metabolic activity. This is due to the increased amino acid reserve. Inflammation, infection, renal failure, and respiratory dysfunctions lead to muscle wasting and muscle atrophy, with severe consequences on the clinical outcome of these patients. Recently, a series of biochemical connections have been observed between the microRNAs and the muscle wasting processes that take place due to inflammation and hypermetabolism. Practically, it has been proven that muscle atrophy is a biochemically active process that is controlled by a series of genetic signals specific for the microRNAs. Soares et al. determined the microRNAs expression under the circumstances of muscle waste in a catabolic profile and an increased activity for microRNA-206 and microRNA-21 were discovered following this study [77,78,79,80,81,82,83,84,85,86,87,88,89,90,91,92,93,94,95,96,97,98,99,100,109,110].

The critically ill polytrauma patient is characterized by severe hypoxia that affects a series of biochemical and metabolic systems. A very important element for this situation is the factor, alpha-1 inducible hypoxia (HIF-1 α). Under conditions of stress and hypoxia, HIF-1α is used as a transcription substrate for a series of genes that modulate cellular metabolism, cellular motility, and angiogenesis. Recent studies have proven the existence of biochemical links between microRNAs and the HIF-1α activity. In this manner, they have identified changes in the microRNAs expression that were closely correlated with the hypermetabolic status, such as microRNA-140 (a genetic regulation reaction specific for chondrocyte), microRNA-140 and let-7 (a specific regeneration reactions for skeletal muscles), microRNA-199a (differentiation reactions for chondrocytes through the SMAD1 transcription factor), and microRNA-365 (proliferation reactions of chondrocytes through histone deacetylase). Another study regarding the expression of regulatory T-lymphocytes and their interaction with microRNAs under metabolic stress showed that in under-nutrition conditions, the expression of transforming growth factor beta 1 (TGF-β1) is altered. Moreover, a series of interactions have been identified between the TGF-β1 and microRNA-29a, microRNA-146a, microRNA-21, microRNA-181a, microRNA-181c, and microRNA-155 [78,79,80,81,82,83,84,85,86,87,88,89,90,91,92,93,94,95,96,97,98,99,100,101,102,103,104,105,106,107,108,109,110,111,112].

## 5. Mathematical Formulas for Predicting Energy Requirements

In the past, there were several mathematical formulas capable of estimating energy needs. After numerous studies, however, it has clearly been proven that these formulas are, in fact, not capable of correct estimations of energy requirements for critically ill patients, especially for polytrauma patients. This is not surprising, as there is no correlation between the mathematical factors included in the formulas and clinical dynamics of these patients [75,76,77,78,79].

In such circumstances, calculating the energy expenditure is important in order to accurately determine the number of calories they need. During the early phases of critical illness, the number of calories consumed is lower than the energy expenditure because the body utilizes the inhabitable glucose. For this reason, there is a risk of overfeeding. In a similar manner, more calories are needed when the critical illness is subsiding, and therefore there is a risk of underfeeding. Significantly, inappropriate energy intake can affect the outcome of the patient during critical illness. To avoid this, energy expenditure estimation methods, such as predictive equations, indirect calorimetry, use of double-labeled water, and reference methods have been applied [77]. All of these methods have disadvantages and advantages, however, preference for one over the other depends on the method with advantages that outweigh the disadvantages. Some research has pointed out that predictive equations tend to be inaccurate most of the time, whereas indirect calorimetry presents a cumbersome setup, including problems with storage of equipment as well as technical limitations. Often, predictive equations are used because indirect calorimetry is not available for all populations in all institutions [78,79].

Nevertheless, studies have shown that these equations sometimes give inaccurate answers despite the age, gender, or weight [80] (Table 1). On the one hand, for example, in a study performed to validate the results produced by the two methods, indirect calorimetry was found to have a ±10% degree of error for measured energy expenditure. As it is considered the gold standard for measuring energy requirements, these ranges are quite acceptable. The Harris–Benedict equation was used in this experiment and produced inaccurate results, with some overestimates and some underestimates. On the other hand, a predictive equation estimate based on an assumption of relationships between age, height, weight, sex, or minute ventilation has not yet been proven.

## 6. Nutritional Therapy Guided with Indirect Calorimetry

**Figure 2:**
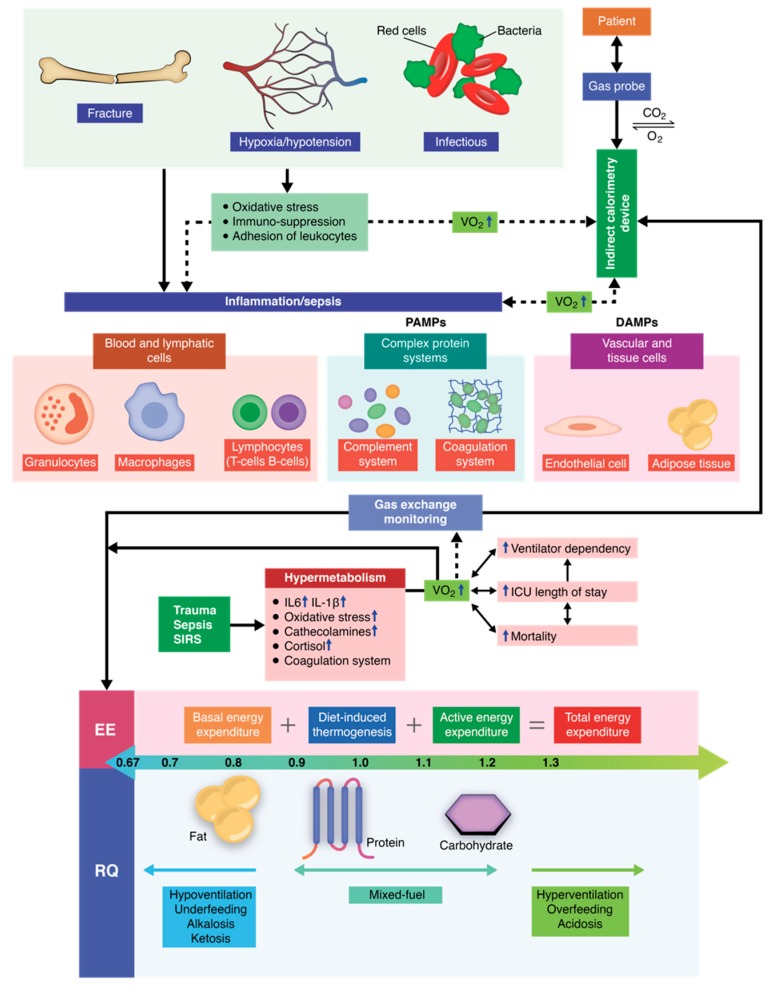
Indirect calorimetry gas exchange monitoring and respiratory quotient interpretation during critically ill conditions.

Indirect calorimetry is one of the most widely discussed methods in the literature, because it is the ideal parameter for determining the energy requirements for critically ill patients who are intubated and mechanically ventilated [89,90]. Although over 90% of critically ill polytrauma patients also present respiratory failure and require prolonged mechanical ventilation, indirect calorimetry can be considered the gold standard for determining appropriate nutritional therapy and adapting the therapy to these specific patients. From a technical point of view, indirect calorimetry is based on real-time monitoring of oxygen consumption (VO_2_) and carbon dioxide production (VCO_2_) [91,92,93]. Clinically speaking, the principle behind indirect calorimetry is represented by the fact that, in the human body, there are no considerable molecular oxygen reserves. The oxygen that reaches the organism is then utilized to produce an energy substrate by oxidizing carbohydrates, protein, and fats. Therefore, we can say that the ratio between carbon dioxide production and oxygen consumption is constant. It has also been proven that the respiratory quotient (RQ) depends on the prevailing type of metabolic oxidation, providing useful information about the energy substrate used at critical moments for these patients [94,95,96]. A simple mathematical formula is used for calculating RQ, represented by the ratio between CO_2_ production and O_2_ consumption (RQ = VCO_2_/VO_2_) [94]. Basically, depending on the oxidized substrate at that point in time, the RQ value will vary at certain intervals, providing supplementary information regarding the metabolic status of these patients (Figure 2).

Numerous studies have shown that a series of complications can arise after inadequate nutritional therapy. Among these are infections, acute kidney injury, acute respiratory distress syndrome, multiple organ dysfunction syndrome and, in the case that the patient survives, late and prolonged recovery [67,97,98]. Another important aspect is the longer ICU stay for these patients. Anbar et al. showed that the time spent in the ICU can be increased by up to three days for patients for whom nutritional therapy is not correctly administered [99].

Regarding the accuracy of the method, there are a series of studies that have shown an increased specificity for indirect calorimetry. Inadomi et al. conducted a study in which they compared oxygen consumption (VO_2_) measured through indirect calorimetry and by the Fick method that uses central venous oxygen saturation (ScVO_2_) and cardiac output (CO) measured by pulse dye densitometry (PDD). This was a prospective study and included mechanically ventilated patients. Following certain tests, they identified major differences between the two studied methods. In the case of indirect calorimetry, VO_2_ was 148 ± 28 mL/min/m^2^ as compared with 110 ± 29 mL/min/m^2^ measured through the Fick method (*p* < 0.01). The authors concluded that, for the measurement of VO_2_, indirect calorimetry remains the gold standard due to its increased accuracy [100].

The use of nutritional therapy, in the case of critically ill polytrauma patients, has grown during recent years because of its implications in the clinical course of these patients, as has been proven in numerous clinical studies. Another important aspect in the development of nutritional therapy, in these particular cases, is represented by the progress of diagnostic methods and by the understanding of the molecular and biological mechanisms involved in the process.

Together with the introduction of artificial nutritional support, a series of complications associated with the nutrition type, be it enteral, parenteral or mixed, have been identified. Several years ago, there were a series of controversies regarding the type of nutrition and caloric input that should be administered to a patient. Therefore, a series of equations have been developed to predict energy requirements in critical patients. Numerous studies have identified a major discrepancy between the values calculated with these formulas and the clinical reality [82,101].

Recently, a new concept has been introduced in the field, a concept of nutrition adapted to the individual needs of each patient, based in particular, on determining energy requirements through indirect calorimetry. In essence, this adapted nutritional therapy is based on the supplementation of specific nutritional deficits by assuring the energy requirements (kcal/day) that the patient can tolerate at that certain critical moment.

It goes without saying that a critically ill polytrauma patient represents a real challenge for the intensive care physician in regard to selecting an approach to nutritional therapy. This is because of the complex immune response, the aggressive pro-oxidative status, and the generalized inflammatory response. In particular, the pathophysiological changes, molecular alterations, and dynamics of inflammatory status modify the clinical status differently from one patient to another, as well energy requirements (kcal/day) and protein turnover are modified continuously, and therefore the estimation of their values through mathematical methods becomes impossible.

With respect to adapting nutritional therapy during the first week from admission to the ICU, in the case of critically ill polytrauma patients, it is recommended that over 50% of their caloric target is administered through enteral feeding. Obviously, if this is not feasible, then, parenteral nutrition is also an option. Another important recommendation is that proteins should not be included in the calculations for the caloric requirements, because, from a biological viewpoint, they are not used in the deposited of muscle mass or the metabolism reactions to cover the energy requirements [76,98,102].

A significant proportion of polytrauma patients is represented by patients with traumatic brain injury (TBI) [2,103,104]. A different aspect, in the case of these patients, is represented by the hemodynamic instability and secondary complications that they develop. From a metabolic point of view, patients with TBI present with a hypermetabolic status, with a specific catabolism regardless of proteins or administered calories. Practically, because of the molecular mechanisms responsible for the links and reactions induced by the cerebral lesions, each patient will have a different degree of catabolism, and therefore different energy requirements. Thus, indirect calorimetry remains the only way to validate the correct determination of energy requirements (kcal/day) for this type of patient. From a biochemical point of view, one of the main causes of accelerated hypermetabolism in these patients is the augmentation of the damage-associated molecular pattern (DAMP). Among these, are the excessive production of cytokines (IL-6, IL-8, and TNF-α) that also induce the excess production of catecholamines, cortisol, and glucagon. Clinically, through the accumulation of complex molecular mechanisms, the energy requirements (kcal/day) of critically ill polytrauma patients with TBI increase significantly and change from day to day under the action of a series of DAMP-specific factors [105].

Allingstrup et al. carried out a study on the topic of early nutrition versus standard nutritional therapy. Regarding the study design, the authors divided the patients into two groups. One group received nutritional therapy based on values obtained through indirect calorimetry and urinary urea nitrogen, whereas the other group received nutritional therapy based on a recommended administration of 25 kcal/kg/day. The authors randomly enrolled 203 patients in the study, and the study results showed a statistically significant difference regarding negative energy (*p* < 0.001) and protein balance (*p* < 0.001) for patients whose nutritional therapy was based on indirect calorimetry, however, following this study, there were no significant differences regarding mortality or time spent in the ICU and hospital days [101].

In agreement with the recommendations, indirect calorimetry is the gold standard for invasively mechanically ventilated patients, however, recently, indirect calorimetry was also tested as a method for application in patients ventilated non-invasively. Siirala et al. conducted a study regarding the accuracy of indirect calorimetry in the case of non-invasive mechanically ventilated patients as compared with patients with spontaneous breathing and they could not identify any significant differences regarding the resting energy expenditure (REE) values and RQ in the two groups [91].

Sunderland et al. carried out a study including critical patients with TBI, in which they compared the measured energy expenditure through indirect calorimetry to the energy requirements calculated using different mathematical formulas. They included 102 patients in the study, with over 385 measurements. They showed an increased accuracy for indirect calorimetry, validating once again the clinical accuracy of the method [106]. Another study carried out by Maxwell et al. supported these results, proving that indirect calorimetry is the gold standard for determining energy requirements (kcal/day) in critical patients [107].

A revolutionary study was conducted by Strack et al. on the clinical outcomes and implications of indirect calorimetry-guided nutritional therapy. They proved, when guided by indirect calorimetry, that nutritional therapy and the administration of a minimum of 1.2 g proteins/kg/day brings significant improvements in survival rates after 28 days in the ICU. An important aspect of this study was that they did not identified any significant differences regarding mortality in the case of men [108].

In addition, the critically ill polytrauma patient is characterized by a series of pathologies and imbalances, all of which are interconnected and have accelerated dynamics. These rapid pathophysiological and metabolic changes lead to an extremely dynamic energy expenditure profile that changes on a daily basis [102,103,104,105,106,107]. On the one hand, the most common changes that lead to an increased energy expenditure (kcal/24 h) are represented by hyperventilation, hyperthermia, overfeeding, infections, inflammation, metabolic acidosis, hyperthyroidism, and pheochromocytoma. On the other hand, there are certain pathological situations responsible for decreased energy expenditure (EE, kcal/24 h) such as metabolic alkalosis, underfeeding, hypoventilation, sedation and coma, hypothermia, and hypothyroidism. Moreover, certain pathologies exist that are able to dynamically modify the energy expenditure, one of which is cancer that dynamically increases EE (kcal/day) because of inflammation and aggressive cellular division. Other such pathologies are chronic kidney disease and diabetes which both, through inflammation and metabolic acidosis, are responsible for an increased metabolic activity and lead to increased EE (kcal/day) [105,106,107,108].

RQ is another important aspect when using indirect calorimetry. It can be obtained directly by monitoring respiratory gases and can guide nutritional therapy in a patient-centered, individualized manner. The complexity of nutritional substrate biochemical and metabolic oxidation processes makes adapting nutrition for each particular segment impossible. Six O_2_ moles are needed for the oxidation of glucose, with the further production of six moles of CO_2_ for each mole (180 g) of glucose. In this particular situation, the RQ (VCO_2_/VO_2_) equals one. Through a similar mechanism, the oxidation of one mole of fat (palmitoyl–stearoyl–oleoyl–glycerol), there is a consumption of 78 moles of O_2_ and a production of 55 moles of CO_2_ for each mole of fat. Therefore, the RQ for fat (VCO_2_/VO_2_) will be 0.7. Other studies have also presented similar RQ values following fat metabolism, such as RQ for tripalmitin (fat) 0.71, oleic acid (fat) 0.71, and triolein (fat) 0.7 [99,100,101,102,103,104,105,106,107,108,109,110].

In the case of proteins, the calculations are based on empirical formulas using the urinary nitrogen excretion. Studies have reported that from the metabolism of 6.25 g of protein, 1 g of urinary nitrogen will be produced. In the context of the critically ill patient, the metabolic processes are more complex and interfere with one another, leading to important dynamic changes in RW, such as oxidation of glucose to fat, metabolism of lactate, and reactions involved in ATP production. An example is the transformation of glucose into fat that needs the intervention of pyruvate and acetyl-CoA. Basically, through this complex mechanism, 27 moles of glucose (the equivalent of 4.865 g) will consume six moles of O_2_ (the equivalent of 134 L) in order to produce six moles of fat and 52 moles of CO_2_ (1165 L CO_2_). The biosynthesis and oxidation of lactate is another important process, which is also very specific to the critically ill patient. The mechanisms involved in lactate metabolism are represented by gluconeogenesis and oxidation. For the oxidation of one mole of lactate, the body uses three moles of O_2_ with the production of three moles of CO_2_, however, one mole of CO_2_ will be converted to bicarbonate due to the intervention of a proton (H^+^) in the oxidation process of lactate [113,114].

Jeon et al. conducted a study on 215 adult patients with severe burns focusing on EE using IC in comparison with mathematical equations and proved a high accuracy for measurements performed using IC as compared with the results obtained when solely using equations. They also showed rapid changes in EE that were only detected by IC, proving that the adjustment of nutritional therapy based on mathematical equations is impossible [115].

Zusman et al. developed a similar study regarding the differences between measured EE and calculated EE including 1440 patients. They concluded that equations have low accuracy as compared with indirect calorimetry and cannot replace this method in guiding nutritional therapy [116]. The study by Kreymann et al., on EE changes and their correlation with sepsis and septic shock, showed that the mean VO_2_ in patients with sepsis was 180 ± 19 mL/min/m^2^, whereas the value for patients in septic shock was 120 ± 27 mL/min/m^2^ (*p* < 0.001). Statistically significant changes (*p* < 0.01) were reported regarding the mean resting metabolic rate in sepsis (+55% ± 14%) and in septic shock (+2% ± 24%). The O_2_ extraction capacity was also studied, with maximum values being reported for sepsis as compared with septic shock (VO_2_/DO_2_, 0.39 vs. 0.29, *p* < 0.05). An increase in the resting metabolic rate with +61% ± 21% during recovery from sepsis and septic shock was also proven. Following their study, the research group underlined the importance of IC monitoring for detecting dynamic metabolic changes, as well as their association with the clinical context and with guiding nutritional therapy based on each patient’s hypermetabolic status [98]. Singer et al. reported, from the tight calorie control study (TICACOS), that in-hospital mortality can be reduced when administering nutritional therapy based on IC. They included 130 mechanically ventilated patients in their study that had been divided into two study groups (nutritional therapy based on IC vs. 25 kcal/kg/day). The monitored EE was much higher than the calculated EE (2086 ± 460 kcal/day vs. 1480 ± 356 kcal/day, *p* = 0.01). The group also highlighted a statistically significant difference in the amount of protein administered per day (76 ± 16 g/day vs. 53 ± 16 g/day, *p* = 0.05) [117]. Heidegger et al. carried out a similar study and showed a decrease in the incidence of nosocomial infections in patients that benefited from IC guided nutritional therapy [118].

The study by Tamura et al. investigated REE in cardiac surgery patients as compared with the REE values obtained by IC and the Harris–Benedict equations. The study included 47 patients and demonstrated significant differences between calculated and measured EE values. They also showed that EE calculated by using the Harris–Benedict equation was 1.14 higher than the EE monitored through IC [119]. Dias Rodrigues et al. compared the results of IC with the mathematical equations, in a group of elderly patients, on hemodialysis and reported an overestimation of energy requirements when basing calculations on equations. Their results showed statistically significant differences (*p* < 0.05) between measured EE and the Harris–Benedict method (1339 ± 245 kcal/day), WHO (1385 ± 225 kcal/day), as well as Schofield (1358 ± 203 kcal/day). While investigating the accuracy, they reported a lower accuracy for equations as compared with IC [120]. In a similar study, Valainathan et al. compared the difference in EE values between IC, the Harris–Benedict equation, and a modified Harris–Benedict equation in patients with severe acute pancreatitis. Following this study, they concluded that the Harris–Benedict equation underestimates EE, while the modified Harris–Benedict equation overestimated EE in this patient group, and therefore IC is the most reliable method for calculating the energy requirements [121].

## 7. Nutritional Therapy Guided with Indirect Calorimetry in Critically Ill Pediatric Patients

When looking at the pediatric critically ill population, the energy requirements are of utmost importance. In the first two years of life, the nutritional status has a significant impact on the development of all biological and morphological structures. In preterm newborns, it has been observed that the nutritional status has important implications in organ development, especially due to organ immaturity and low nutritional reserve [122]. A high percentage of newborns also have a diaphragmatic hernia. One of the major risks in this situation is the development of growth failure due to an imbalance between calorie intake and increased catabolic stress. In this patient category, low calorie intake is mainly due to specific organic illnesses, such as gastro-esophageal reflux, oral aversion, and esophageal dysmotility. Moreover, metabolic and surgical stress leads to an underestimation of the energy requirements, with IC being the only method that can be used for a correct appreciation of the nutritional therapy. Haliburton et al. carried out a study on determining EE in infants with a congenital diaphragmatic hernia by using IC, as well as comparing their results with classical formulas. They showed that, after IC monitoring, the energy requirement was 58 ± 18 kcal/kg/day, which was considerably higher than the calculated EE of 46.6 ± 3 kcal/kg/day (*p* < 0.05). This study also showed that 59% of the patients were hypermetabolic with a measured EE of >110% than the predicted EE [122]. Howell et al. also reported significant differences between the metabolic statuses of infants with congenital diaphragmatic hernias when measuring EE with IC as compared with calculated EE [123]. The study by White et al. also supported these results by comparing EE measured through IC and EE calculated with equations such as Schofield (mean % difference, 21.2%), WHO (mean % difference, 23.39%), and White (mean % difference, 36.45%) [124].

Vazquez Martinez et al. also carried out a study on EE in the critically ill pediatric patient. They compared EE from IC and from mathematical equations for 43 mechanically ventilated pediatric patients. They showed a statistically significant difference regarding measurement accuracy, as follows: IC vs. Harris–Benedict (mean differences, 162.9 ± 236.5, *p* = 0.001); IC vs. Schofield (96.74 ± 186, *p* = 0.01); IC vs. Maffies (181.4 ± 232.0, *p* < 0.0001); IC vs. Kleiber (−130.5 ± 178.9, *p* = 0.001); IC vs. Dreyer (296.5 ± 219, *p* < 0.0001); and IC vs. Hunter (−317.7 ± 180.5, *p* = 0.001) [125]. Suman et al. studied the critically ill patient with burns, and also showed significant differences between measured and calculated EE as follows: IC vs. Schofield-HW (mean difference, −64.7 to −22.4 kcal/day); IC vs. Harris–Benedict (mean difference, 640 ± 555 kcal/day); and IC vs. Food and Agriculture Organization, World Health Organization, and the United Nations University (FAO/WHO/UNU) (mean difference, 652 ± 559 kcal/day) [126]. Bott et al. reported similar results following a study that included 52 pediatric patients with bronchopulmonary dysplasia as follows: IC vs Harris–Benedict (mean difference, −15 ± 33.3); IC vs. Schofield-W (−51.3 to −2.0), Schofield-HW (−67.7 to −22.4); and IC vs. FAO/WHO/UNU (−47.5 to 7.4) [127,128].

## 8. Conclusions

In conclusion, we can state that each critically ill polytrauma patient is special and unique because of their characteristic hypermetabolic and hyperinflammatory status. The time spent on mechanical ventilation, the multiple tissue injuries, the organ lesions, multiple site infections, and increased oxygen consumption all lead to dynamic changes in energy consumption for each individual patient. The literature shows that mathematical equations, for the most part, are far removed from the clinical reality of the patient, with significant discrepancies between the results. Therefore, indirect calorimetry becomes the gold standard for monitoring the personalized energy requirements of the patients, respecting the individual clinical dynamics.

## Figures and Tables

**Table 1:** Predictive equation for energy expenditure determination (M, male; F, female; RMR, resting metabolic rate; REE, resting energy expenditure; BMR, basal metabolic rate; FFM, fat free mass; FM, fat mass; TBSA, total body surface area; BSA, body surface area; and AF, activity factor, typically 1.2–1.4).

Name	Formula	Accuracy (%)	References
Mifflin St Joer	M: RMR = 9.99 × Weight + 6.26 × Height − 4.92 × Age + 5 F: RMR = 9.99 × Weight + 6.25 × Height − 4.92 × Age − 161	17.8	[12,78,80,81,82,83,84,85,86,87,88]
Harris−Benedict	M: RMR = 66.47 + 13.75 × Weight + 5.0 × Height − 6.75 × Age F: RMR = 655.09 + 9.56 × Weight + 1.84 × Height − 4.67 × Age	31.3	
Owen	M: RMR = 879 + 10.2 × Weight F: RMR = 795 + 7.18 × Weight	48	
Carlson	REE = BMR × [0.89142 + (0.01335 × TBSA)] × BSA × 24 × AF	94	
Curreri	REE = 25 × Weight (kg) + 40 × % BSA burned	91	
Bernstein	REE = 19.02 × FFM + 3.72 × FM − 1.55 × Age + 236.7	19	
Xie	REE = (1000 × BSA) + (25 × TBSA)	91	
Horie–Waitzberg	REE = 560.43 + (5.39 × Weight) + (14.14 × FFM)	65.8	
Ireton-Jones	M: REE = 606 + (9 × Weight) − (12 Age) + 400 (if ventilated) + 1400 F: REE = Weight − (12 × Age) + 400 (if ventilated) + 1444	60	
Muller	REE = 0.05 × Weight + 1.103 × Sex + 0.01586 × Age + 2924	68	
Livingston	M: REE = 293 × Weight ^0.4330^ − 5.92 × Age F: REE = 248 × Weight ^0.4356^ − 5.09 × Age	67	
Schofield W	M: REE = 11.711 × Weight + 587.7 F: REE = 9.082 × Weight + 658.5	59	
Henry	M: REE_60–70y_ = 13 × Weight + 567|REE_≥71y_ = 13.7 × Weight + 481 F: REE_60–70y_ = 10.2 × Weight + 572|REE_≥71y_ = 10 × Weight + 577	66	
De Lorenzo	M: REE = 53.284 × Weight + 20.975 × Height − 23.859 × Age + 487 F: REE = 46.322 × Weight + 15.744 × Height − 16.66 × Age − 944	63	
20 Kcal/kg Ratio	REE = Weight × 20	44	
Lazzer	M: REE = 0.048 × Weight + 4.655 × Height − 0.020 × Age − 3.605 F: REE = 0.042 × Weight + 3.619 × Height − 2.678	59	
Korth	REE = 41.5 × Weight + 35.0 × Height + 1107.4 × Sex − 19.1 × Age − 1731.2	63	
Huang	REE = 10.158 × Weight + 3.933 × Height − 1.44 × Age + 273.821 × Sex + 60.655	71	
Weijs	REE = Weight × 14.038 × Height × 4.498 + Sex (1 = M, 0 = F)	48	
Fredrix	REE = 1641 + 10.7 × Weight − 9 × Age − 203 × Sex	70	
Cunningham 1989	REE = (21.6 × FFM) + 370	63	
Wang et al.	REE = (21.5 × FFM) + 407	59	
Lurhmann	REE = 3169 + 50.0 × Weight − 15.3 × Age + 746 × Age	58	
Swinamer	REE = (945 × BSA) − (6.4 × Age) + (108 × Temperature) + (24.2 × Respiratory rate) + (817 × V_y_) − 4349	55	
Frankenfield	REE = 925 − (10 × age) + (5 × Weight) + (281 if male) + (292 if trauma present) + (851 if burns present)	28	
Penn State 2003	REE = (0.85 × Value from Harris−Benedict equation) + (175 × T_Max_) + (32 ×V_T_) − 6433	39	
Penn State 1998	REE = (1.1 × Value from Harris–Benedict equation) + (140 × T_Max_) + (32 × V_E_) − 5340	68	
